# Visual and somatosensory information contribute to distortions of the body model

**DOI:** 10.1038/s41598-019-49979-0

**Published:** 2019-09-19

**Authors:** Valeria Peviani, Lucia Melloni, Gabriella Bottini

**Affiliations:** 1 Department of Brain and Behavioural Sciences, University of Pavia, Via Bassi 21, 27100 Pavia, Italy; 2 Department of Neuroscience, Max Planck Institute for Empirical Aesthetics, Grüneburgweg 14, 60322 Frankfurt am Main, Germany; 3 Department of Neurology, New York University School of Medicine, 240 East 38th St 10016, New York, NY USA; 4 Cognitive Neuropsychology Center, ASST Grande Ospedale Metropolitano Niguarda, Piazza dell’Ospedale Maggiore 3, 20162 Milan, Italy; 5 NeuroMi, Milan Center for Neuroscience, Milan, Italy

**Keywords:** Human behaviour, Perception

## Abstract

Distorted representations of the body are observed in healthy individuals as well as in neurological and psychiatric disorders. Distortions of the body model have been attributed to the somatotopic cerebral representation. Recently, it has been demonstrated that visual biases also contribute to those distortions. To better understand the sources of such distortions, we compared the metric representations across five body parts affording different degrees of tactile sensitivity and visual accessibility. We evaluated their perceived dimensions using a Line Length Judgment task. We found that most body parts were underestimated in their dimensions. The estimation error relative to their length was predicted by their tactile acuity, supporting the influence of the cortical somatotopy on the body model. However, tactile acuity did not explain the distortions observed for the width. Visual accessibility in turn does appear to mediate body distortions, as we observed that the dimensions of the dorsal portion of the neck were the only ones accurately perceived. Coherent with the multisensory nature of body representations, we argue that the perceived dimensions of body parts are estimated by integrating visual and somatosensory information, each weighted differently, based on their availability for a given body part and a given spatial dimension.

## Introduction

Knowing the dimensions of the body is critical to plan actions and to navigate through space. Yet, we misestimate both size and shape of our body parts^1–5^, resulting in a decreased accuracy of the motor outcome in the absence of visual corrective cues^6^. The origin of these distortions is currently under intensive debate.

Distortions of perceived body dimensions occur for many body parts^1^, but the bulk of evidence for a distorted body model comes from studies investigating the metric representation of the hand^7^. In proprioceptive hand localization tasks, hand width is typically overestimated and hand length is underestimated. Similarly, tactile stimuli delivered across the hand dorsum are perceived as larger compared to those delivered along the hand^7^. It has been hypothesized^3,8^ that such anisotropy might reflect the presence of oval-shaped receptive fields whose longer axis is oriented along the length of the limbs^9–11^. If this is the case, the distortions of body part representations might be a direct consequence of the somatotopic organization.

Furthermore, distortions of length estimates of body parts scale with the respective body part’s tactile acuity and actual length: less sensitive body parts (e.g., arm, leg and torso) are perceived as longer than more sensitive body parts (the hand and the foot), and longer body parts appear less overestimated then shorter ones^2,4^. Both findings are consistent with an origin of these distortions in somatosensory cortex: tactile distance is perceived as shorter on a body part with a low receptive field density than when applied to one with a high receptive fields density^12^. However, the difference perceived in terms of the tactile distance is not usually matched in magnitude to the difference in receptive field density. According to the “reverse distortion” hypothesis^2^, the metric representation of a certain body part may be overestimated to compensate for its low receptive field density, and therefore to compensate the distorted cortical representation of a specific body part in the somatosensory maps^13,14^.

However, recent studies have called into question whether distortions of perceived body dimensions are (solely) of somatosensory origin. Specifically, it has been demonstrated that the often reported distortions of the hand representations are driven by a mislocalization of the knuckles, which are perceived as closer to the fingertips than they actually are^15–17^. Importantly, the same pattern of distortions as those reported for the hand occur when participants estimate the dimensions of objects that resemble hand shape, e.g., a rake^17^. This suggests that body representations are (also) shaped by non-somatosensory factors that are not specific to the body. One such factor could be inaccurate spatial knowledge of hand structure that arises from a mismatch between the visually perceived end of the finger and the actual anatomical location of the knuckles^17^. However, because Saulton and colleagues^17^ exclusively focused on hand representations, it is possible that their finding is specific to the hand, and that distortions reported for other body parts^1^ are indeed of somatosensory origin.

To better understand the sources of the distortions of the body model we compared the metric representations across body parts affording different tactile sensitivity but also different degree of visual accessibility. If the same visual-based pattern of distortions shapes our mental representations, it follows that different body parts should be similarly misrepresented. In contrast, if other factors such as the sensitivity of a body part have an additional impact on its metric representation, we should be able to detect different patterns of distortions across body parts.

**Figure 1:**
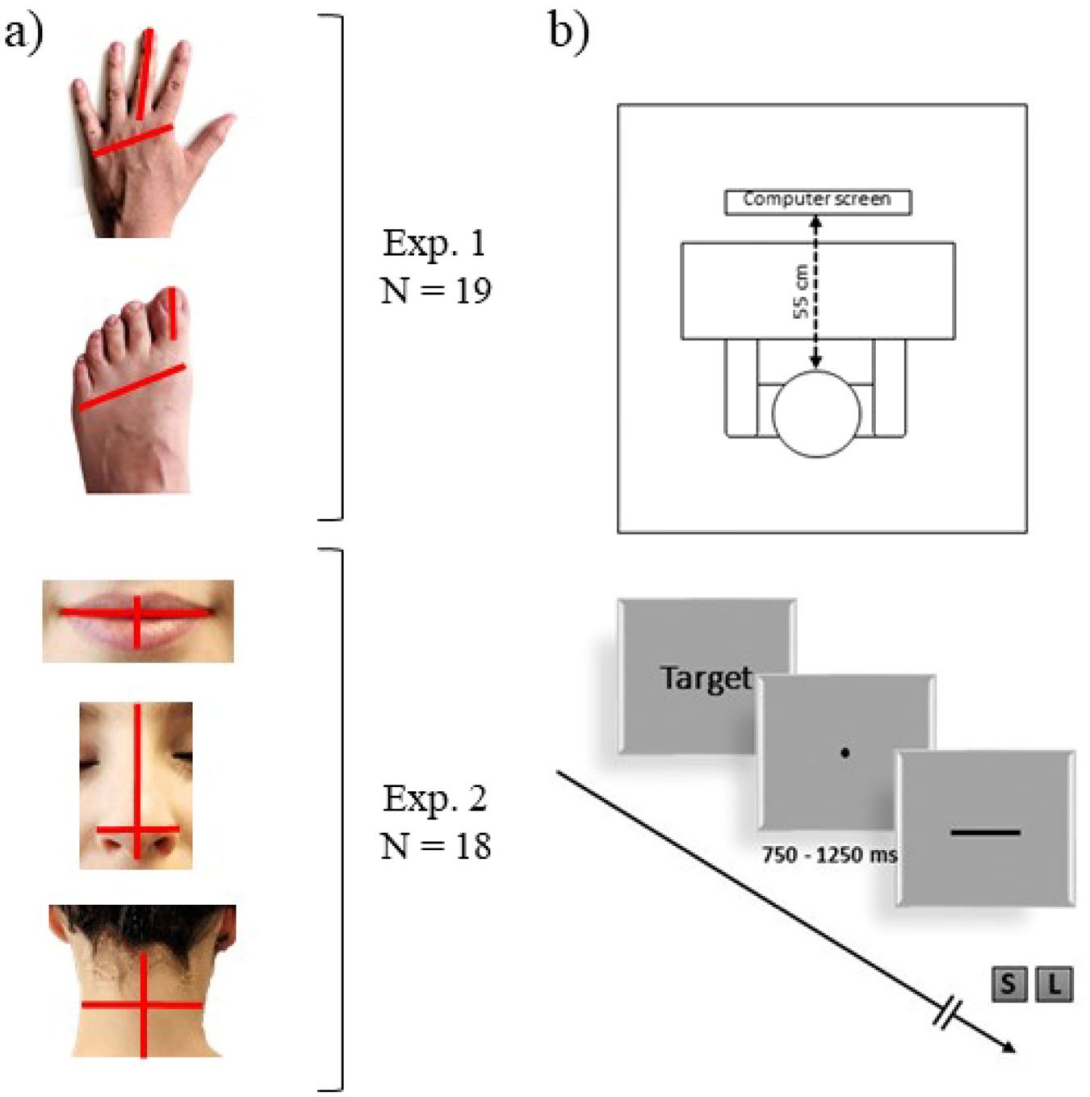
(**a**) Depicts the five body parts studied in experiment 1 and 2. Red lines represent the two dimensions i.e., length and width investigated in the Line Length Judgment task. (**b**) Experimental setup and paradigm.

We thus investigated absolute differences across the representations of two orthogonal dimensions, i.e., width and length, of several body parts (Fig. 1a). In the first experiment, we compared the hand dorsum with that of the foot, as they are characterized by a similar tactile acuity^18^ but have a different aspect ratio. In the second experiment, we compared the representations of the lips, as they are highly sensitive^19–22^ and coupled with the hand during motor planning and interaction with the environment^23,24^; of the nose, for which we have roughly a similar degree of visual access; and of the dorsal portion of the neck, as it has poor sensitivity^25^ and visual accessibility. To evaluate the metric representation across those different body parts we used the Line Length Judgment (LLJ) Task^26^ and asked participants to compare the perceived length and width of the body part with the perceived length of a line segment presented on a computer screen using a two-alternative forced choice task (Fig. 1b).To preview, we found evidence supporting the existence of systematic misestimations of the dimensions of body parts, to which somatosensory and visual factors contribute to differing degrees depending on the body part and dimension considered.

## Results

We first investigated the perceived length and width of the hand and the foot. We found that both the perceived length and width of those body parts are underestimated i.e., they are perceived as shorter and thinner than their actual dimension (HAND length EE = −10.07 ± 6.45%, t(13) = −5.727, p < 0.001, BF_10_ = 349.928; width EE = −5.28 ± 7.72%, t(13) = −2.676, p = 0.019, BF_10_ = 3.367; FOOT length EE = −9.37 ± 9.99%, t(13) = −4.780, p < 0.001, BF_10_ = 94.385; width EE = −5.23 ± 7.47%, t(13) = −3.525, p = 0.004, BF_10_ = 12.846). Despite the differences in actual size across the hand and the foot, both body parts appear to be similarly distorted (main effect of body part: F(1,13) = 0.317, p = 0.583, BF_10_ = 0.247, main effect of dimension: F(1,13) = 3.603, p = 0.080, BF_10_ = 1.329, interaction: F(1,13) = 0.008, p = 0.930, BF_10_ = 0.200).

We next investigated the perceived dimensions of the lips, the nose and the neck, which vary in tactile acuity and visual accessibility. The lips and the nose were underestimated in their length (LIPS, EE = −7.56 ± 8.52%, t(15) = −3.321, p = 0.005, BF_10_ = 10.340; NOSE, EE = −5.57 ± 8.88%, t(15) = −3.94, p = 0.001, BF_10_ = 33.345) and also in their width (LIPS, EE = −3.20 ± 6.95%, t(15) = −2.243, p = 0.040, BF_10_ = 1.773; NOSE EE = −14.45 ± 8.02%, t(15) = −10.410, p < 0.001, BF_10_ = 440e + 6). In contrast, the length and width of the neck were accurately perceived (length EE = 0.79% ± 9.80, t(15) = 0.323, p = 0.751, BF_10_ = 0.268; width EE = −3.06 ± 7.80%, t(15) = −1.571, p = 0.137, BF_10_ = 0.709). We evaluated the effect of body part and dimension on the EE in a 2 × 3 within-subject Analysis of Variance (ANOVA). We found a main effect of dimension (F(1,15) = 5.272, p = 0.037, BF_10_ = 10.73), reflecting a bigger underestimation of the perceived length compared to the perceived width, a significant main effect of body part (F(1,15) = 4.594, p = 0.049, BF_10_ = 17.023e + 4) and a significant interaction (F(1,15) = 4.755, p = 0.046, BF_10_ = 24.950). Post-hoc tests revealed that the perceived length of the lips and nose were underestimated more compared to the neck (lips vs neck length EE: p = 0.049, BF = 3.681; nose vs neck length EE: p = 0.034, BF = 4.993). In contrast, the perceived width of the nose was more underestimated than those of the lips and of the neck (nose vs lips width EE: p < 0.001, BF_10_ = 1414.796; nose vs neck width EE: p = 0.001, BF_10_ = 180.708).

**Figure 2:**
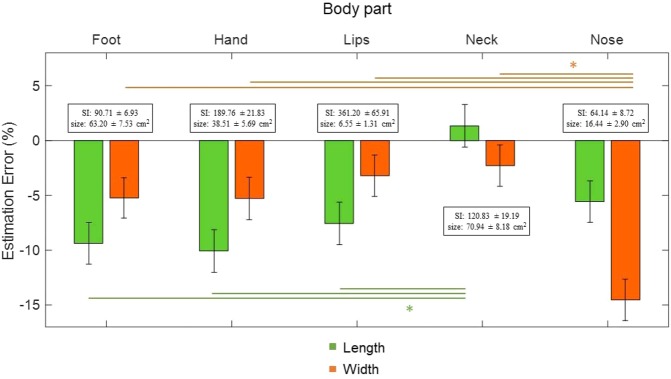
Body part estimation error (EE: 100* perceived size/actual size) for 5 different body parts. Bars depict the mean estimation error and standard error of the mean for length (green) and width (orange). The inset above each body part denotes the actual Shape Index (SI: 100* body part width/body part length) and Size (body part length*body part width). Asterisks denote significance at p < 0.05, in post-hoc tests Bonferroni corrected for multiple comparisons.

To compare the pattern of distortions in the perception of the length and width across different body parts we pooled the data from the two experiments. We fitted a Linear Mixed-Effects Model with body part and dimension as fixed effects, incorporating random intercepts and slopes to account for inter-subject variability. We confirmed that the residuals were normally distributed (Shapiro-Wilk test of normality: p = 0.238). Figure 2 shows the error estimation for both dimensions and body parts. We found a significant effect of body part (F(4,177) = 7.124, p < 0.001), an interaction between body part and dimension (F(4,177) = 5.291, p < 0.001) and no main effect of dimension (F(4,177) = 0.14, p = 0.906). Post-hoc tests revealed that the length of the neck was more veridically perceived (less underestimated) than the length of the hand (p < 0.001), the foot (p = 0.001) and the lips (p = 0.014); whereas the perceived width of the nose was significantly more distorted (underestimated) than that of all other body parts (nose vs hand: p = 0.008, nose vs foot: p = 0.005, nose vs lips: p < 0.001, nose vs neck: p < 0.001).

**Figure 3:**
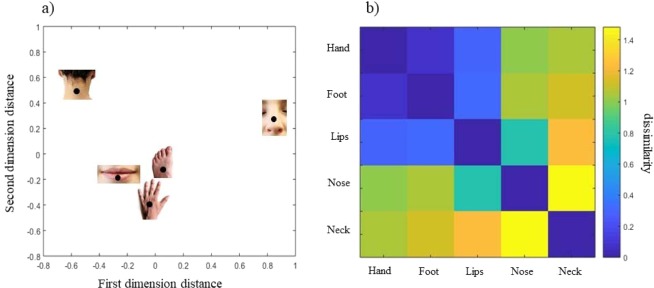
(**a**) Multidimensional scaling on the estimation errors (EE) for the length, the width, the shape (width*length/100) and the size (width*length) is projected onto two dimensions. (**b)** Dissimilarity matrix across body parts. Proximity in the 2D space and blue colors in the dissimilarity matrix indicate higher similarity.

To further study and graphically describe how the representations relate to one another, we used multidimensional scaling. Starting from the Euclidean distances among the EEs referred to the body parts’ length and width dimension, shape (width*length/100) and size (width*length), the multidimensional scaling can be projected to two dimensions (Fig. 3a), and depicted as a dissimilarity matrix (Fig. 3b). The numerical dissimilarity matrix is available in the Supplementary Information (1). This analysis revealed that body parts cluster in three separable groups: a cluster with high similarity between the perceived size of the mouth, hand, foot and lips, which is differentiable from the representations of the neck and the nose. Specifically, hand, foot and lips representations are alike, as suggested by their closeness in Fig. 3a and the dark blue cluster signaling high similarity in Fig. 3b. Instead, both the representation of the neck and that of the nose are isolated and characterized by a distinct pattern of distortions.

**Figure 4:**
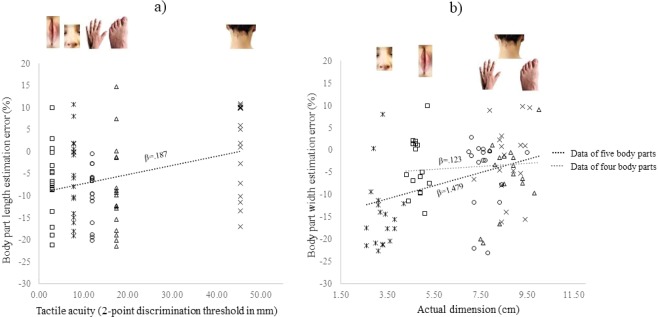
Best-fitting models predicting the EEs for length (**a**) and width (**b**) across five body parts. (**a**) The two-point discrimination threshold (expressed in mm) is plotted against the body part length EE for each subject and body part. Black dashed line depicts the slope relating the length EE to the tactile acuity of the five body parts. (**b**) The actual width is plotted against the width EE of each body part. Black dashed line depicts the slope relating actual size to the width EE for all body parts. Grey dashed line depicts the slope excluding the nose data.

The body parts tested in our study differ in their proportions: some body parts are larger in their horizontal than in their vertical dimension e.g., the lips; while others have the opposite pattern i.e., they are larger in the vertical than in the horizontal dimension, e.g., the nose. They also differ in their tactile acuity. For instance, two tactile stimuli separated by a small distance can be detected on the lips, while two stimuli on the neck need to be at much larger distances to be discerned. We reasoned that those factors or a combination thereof could underlie the pattern of distortions observed across different body parts. To test this hypothesis, we fitted four Linear Mixed-Effects Models testing for the effect of the actual size of the body parts in their vertical and horizontal dimensions, their respective tactile acuity, and their combined effect on each dependent variable (EE for length and width). Each model, along with the fixed intercept and slopes, incorporated random intercepts and slopes to account for inter-subject variability. We also included a null model. For all fitted models the residual distributions were checked through QQ-plots to ascertain the normality assumption. For the EE length, we found that tactile acuity explained a significant proportion of the variance (Likelihood ratio = 13.580, EE (t(47) = 3.117, p = 0.003, with β = 0.187), while the size of the body part or its combined effect with tactile acuity did not improve the fitting (Fig. 4a). This means that the lower is the tactile acuity, the more accurate is the length estimation. Regarding the EE on the width, the best model was the one considering the actual size of the body part (Likelihood ratio = 13.757, p = 0.003: t(47) = 3.840, p < 0.001, with β = 1.479). Introducing tactile acuity and combining it with the actual size of the body part did not further improve the fitting. Visual inspection of the scatterplot (Fig. 4b) suggested that the EE related to the nose width could be contributing to the pattern of results. The nose width - the body part with the smallest wide dimension investigated - was also unexpectedly highly underestimated, compared to the other body parts. We therefore fitted the Linear Mixed-Effects Models considering the four remaining body parts. When removing the nose from the models we observed that the effect of the actual size of the body part was no longer significant (t(30) = 0.238, p = 0.813, with β = 0.123) and the fitting did not improve (Likelihood ratio = 0.238, p = 0.971). The full set of results from the different models is reported in the Supplementary Information (2).

## Discussion

We set out to test whether distortions in the representations of one’s own body parts arise from somatosensory or non-somatosensory, e.g., visual, factors. We found that the answer is not a simple “either, or”, but that both somatosensory and visual factors contribute to the misestimation of the dimensions of body parts. Specifically, we found that the perceived dimensions of the hand, of the foot, of the lips and partially of the nose are similarly underestimated, despite their different physical size and tactile acuity, suggesting that a systematic metric bias is affecting mental representations. On the contrary, the dorsal portion of the neck is accurately estimated in its dimension, suggesting that such metric bias might arise when visual information is weighted more in building up the mental representation. Furthermore, we found evidence supporting the existence of different mechanisms underlying the estimation of the length and of the width of the body parts considered. While the length Estimation Error (EE) is predicted by the tactile acuity, the width EE is not, again pointing at the different weight of somatosensory and visual information in estimating distances between the horizontal boundaries versus the vertical ones. Together, this suggests that we access multisensory, possibly higher-level representations when estimating our own body parts, at least in the processes underpinning the tasks performed in this study.

Previous studies have hypothesized that distortions in the body model arise from the somatosensory cerebral representation (homunculus)^2–4^. At first sight, our data are broadly consistent with this view: first, our data suggest that tactile acuity, measured as two-point discrimination threshold, influences the EE when the vertical dimension is considered. This can be predicted by the reverse distortion hypothesis^2^, which posits that differences in the tactile acuity are balanced by differences in the metric mental representations. The reverse distortion hypothesis further predicts that, given an equal degree of sensitivity, body parts that are physically larger are less distorted, meaning that the tactile acuity should better explain the size misestimation when scaled by the actual size of the body part. This is, however, not what we found. Instead, we found that the combined effect between the body part’s actual dimension and tactile acuity does not better predict the length EE. It is nevertheless conceivable that the smaller amount of variation in size of the body parts we considered (on the order of few centimeters) made the effect of the actual dimension difficult to detect.

As we measured the perceived dimensions of body parts along two orthogonal axes (medio-lateral dimension, or “width”, and rostro-caudal dimension, or “length”), we could further show that the effect of tactile acuity on the perceived dimensions of a body part depends on whether the length or the width of the body part is being judged. In fact, neither the tactile acuity alone nor its combined effect with the actual width was effectively explaining the variance of the width EEs. This and results by Stone and colleagues^5^ on leg representations speak against a purely somatosensory origin of the distortions, because following the reverse distortion hypothesis, the representation of the body metric would need to compensate for the homunculus distortions in its longitudinal as well as transversal components.

Instead, our results also point to a visual influence on body representations. While the medio-lateral boundaries of the body parts considered in the present study were all clearly defined in visual terms, the proximo-distal boundaries were less clearly defined for the hand (tip and knuckle of the middle finger), foot (tip and knuckle of the toe) and neck (upper and higher boundary). This expands the results of Saulton and colleagues^17^, who focused on hand representations, to several other body parts.

Furthermore, we found a similar pattern of distortions for the lips as that observed for the hand and for the foot (Figs 2 and 3a,b), despite major differences in size and tactile acuity between those body parts. This finding further suggests that the size or aspect ratio of the body parts is unlikely to explain the pattern of distortion in the body model. A particularly striking finding is that the dorsal portion of the neck was the only body part among those tested to be represented accurately in its dimensions. If our body representation is affected by a systematic metric bias, such that body parts are underestimated in their dimensions, the neck should have been perceived similarly to other body parts, which was clearly not the case. Visual inputs are undoubtedly more relevant for building up and storing the representations of the hand, lips and foot, whereas it is unlikely that the representation of the dorsal portion of the neck has been built upon visual inputs. No significant variation across the perceived dimensions of the hand, foot and lips was observed, despite that their degree of visual exposure may also vary. Consequently, it can be argued that what drives the effect is an all-or-none cumulative exposure over time, which is virtually absent for the dorsal portion of the neck, while comparatively high for the hand, foot and lips. Such striking differences possibly underlay the observed patterns of responses in terms of perceived dimensions.

Somewhat unexpectedly, we observed that the nose width was more underestimated than the width of all other body parts (while the estimation of the nose length was also similar to that of the lips, hand and foot). Given the small size of the nose width, it could be argued that an underestimation of a few millimeters is amplified resulting in a relatively high EE. However, if the size of the body part played a role, we should have obtained similar effects for body parts with small sizes such as the height of the lips or the length of the toe, which was not the case. Another possible explanation may be rooted in the intrinsic shape of the nose. Among all the distances considered, the nose width is probably the most difficult to be retrieved in 2D-space, as the LLJ task required. In fact, the nose can be represented as an irregular tetrahedron, with one of the vertices aligned with the nose tip. The task instructions implicitly required the participants to retrieve the 2D-size (in terms of basis and height) of one of the faces of the polygon, the one opposite to the nose tip. While the height of that face is easily projected onto the longitudinal edge of the tetrahedron, the basis cannot be projected onto an edge, possibly increasing the difficulty of its estimation.

It is interesting to note that our results regarding the perceived hand dimensions apparently diverge from those reported in the literature. A previous study reported an underestimation up to 40% of the distance between the tip and the knuckle of the middle finger^16^, using a localization task. However, when using the LLJ task in the same group of participants such distance was underestimated by only 25%. The discrepancies in the degree of the misestimation have been attributed to the fact that the LLJ task appears to rely less on a somatosensory representation and more on the visual body image, which is more accurate^16,26^. Furthermore, when participants were asked to refer to the distance between the middle fingertip and its basis rather than its knuckle, avoiding biases related to the mislocation of the finger knuckles, the underestimation decreased from 40% to about 30%^17^. We argue that our results, and in particular, the degree of the misestimation we obtained, are comparable to those that were previously reported when using the LLJ task. Similarly, in previous studies, the hand width (i.e., distance between the knuckles of the index and little fingers) was overestimated up to 80% using a localization task^8,26^, but dropped to about 7–10% when assessed through the LLJ task^16,26^. Importantly, when participants were asked to estimate the width of their hand referring to the distance between the bases of the little and index fingers, it was perceived as slightly underestimated (around 4–5%^17^). This is a range comparable to the one we obtained in our study.

As our sample mostly included female participants, we were not able to evaluate whether the pattern of distortions in the body representation varies as a function of gender. However, a recent meta-analysis of 19 experiments assessing the hand metric representation reported comparable estimates across genders when differences in the absolute size of the actual hand across genders were controlled for. Specifically, participants with bigger hands tended to perceive their hands as smaller compared to participants with smaller hands, indicating a form of regression to the mean^27^. Assuming that similar effects apply to estimates of other body parts, it is conceivable that gender does not moderate the perceived dimensions of the body parts. Future research introducing higher variability in the actual size of the body and testing a large gender-balanced sample is needed to shed light into this issue. Along the same lines, in our study we have relied on estimates of tactile sensitivity extracted from the literature, future investigations could better characterize the relationship between the tactile acuity of a body part and its perceived dimensions by systematically evaluating in each participant the two-points discrimination threshold, as well as other parameters such as the tactile sensitivity. This methodological optimization would extend our results as well as those reported by previous research^2^.

An interesting question that deserves attention relates to whether the size in visual angle of a body part has an influence on its mental representation. Specifically, whether the distance between a body part and the eyes of its owner influences the metric properties of the mental representation of that body part. Under this scenario, body parts that are farther away from the eyes (e.g. the foot) would be more underestimated than body parts that are closer to the eyes (e.g. the lips). We consider this unlikely given that the distance of each body part under investigation from the eyes is highly variable, and thus the degrees of visual angle are constantly changing with posture, age, etc. For instance, the distance from our feet, and therefore their size in degrees of visual angle, continuously varies across postures and over time. Similarly, the position of our hands with respect to our head is highly variable, and it possibly varies depending on many other factors, such as age (i.e. newborns often bring their hands very close to their head) and lifestyle (i.e. doing computer work versus working in a building site). Analogously, the size in degrees of visual angle of objects we see in the outside world varies enormously depending on our distance from them. How the visual system resolves such variability is still a topic of investigation^28,29^. The size-depth invariance principle, for which the ratio between the perceived size and distance of an object is approximately constant^30,31^, has been proposed to regulate absolute size perception^32^. Binocular information, such as the interocular distance, also comes into play when size is estimated^28^. Conceivably, the same phenomenon of size constancy occurs when body parts are involved, allowing for an invariant representation of their size which is also functionally relevant in everyday interaction with the environment and the objects in it. Furthermore, since we are exposed continuously to the sight of our body parts, which can be at a limited range of possible distances, their perceived absolute size might be even more precise compared to objects. Indeed, familiarity plays a role in the accuracy of the size judgment of an object, especially when such an object is only described verbally^33^, which is the case inour experiments.

However, anisotropic effects influencing some attributes of visual perception have been described. For instance, there is a tendency to perceive the upper half of the visual space as larger than the lower half^34^. In regards to size estimation, it might be asked whether the perceived size of a body part is influenced by its spatial location - not just its distance - relative to the oberver’s eyes. Further studies are needed to investigate the possible effect of distance and spatial location of an object on its size estimation, and to test how such factors might have a role in the estimation of body parts’ dimensions. For instance, participants could be asked to estimate the dimensions of an object at different distances and spatial locations, resembling the typical distance and spatial locations of different body parts (e.g. hand, foot). By applying such experimental manipulation, it would be possible to exclude that the difference in size estimation across body parts is due to asymmetries in size estimation of three-dimensional objects in the near space.

Taken together, our results support previous findings of systematic distortions of the dimensions of body parts. Both somatosensory components, i.e., tactile acuity and visual components, i.e., the demarcation of visual landmarks, seem to contribute to these distortions. The pattern of size misestimation is similar among body parts which are visually accessible, but strikingly different for a body part which is not (the dorsal portion of the neck). This suggests that the two (and possibly other) sources of information are weighted to form a multisensory body representation depending on their respective availability. For instance, it is plausible that visual inputs are weighted more than the somatosensory ones for the visually accessible body parts. Similarly, at least for the body parts assessed in our experiments, the visual bias might be more relevant in building up the representation along the medio-lateral axis, compared to the proximo-distal one, as overall the medio-lateral boundaries are less visually defined in our body parts sample. As a matter of fact, we showed that the tactile acuity significantly predicts the misestimation of the proximo-distal dimension of body parts whereas it does not have an effect on the medio-lateral estimation. Multisensory representations of the body are formed in parietal cortex^35^ and/or through interactions between unisensory processing centers: for example, it has been shown that visual information modulates somatosensory processing^36^ and conversely, that somatosensory information affects visual processing^37^.

Within the framework of body representations, our findings highlight that considering the relative weight of incoming and stored information from multiple senses is extremely relevant when studying how the body is represented. We argue that, in a future perspective, it may be worth further exploring how the multisensory integration process underlying body representations works for different body parts, given their diversity in terms of sensitivity, movability, and spatial relations relative to each other. Overall, our results strongly support the notion that body representations are built from the integration of multiple sensory inputs available in a particular moment^38^.

## Methods

### Participants

Nineteen (17 females) participants took part in study 1 (mean age: 23.70 ± 6.03). Eighteen female participants took part in study 2 (mean age 24.00 ± 5.98). All participants were right-handed according to the Edinburgh Handedness Inventory^39^, with a mean laterality index of 92.27 ± 6.20 in the first experiment and of 95.11 ± 7.12 in the second experiment. Participants received university credits or monetary compensation for their participation. Informed consent was obtained prior to each study. Both studies received approval from the local ethical committees (University of Pavia and Ethics Council of the Max Planck Society) and adhered to the ethical standards of the Declaration of Helsinki.

### Task and procedure

We evaluated the metric representation of five different body parts by means of a Line Length Judgment (LLJ) task adapted from a previous study^26^ (Fig. 1b). Participants were asked to determine whether the length of vertically or horizontally oriented lines was shorter or longer compared to a specific body part serving as a reference. In study 1, the reference was either the left hand or the left foot of the participant. In study 2, the reference was either the nose, the lips or the dorsal portion of the neck. For each body part, participants had to compare the lines to either the width (medio-lateral dimension) or the length (rostro-caudal dimension) of that body part. To avoid mental rotation, we presented horizontally-oriented lines whenever subjects made a width judgment and vertically-oriented lines whenever subjects made a length judgment. A block started with a written instruction indicating the body part serving as reference and the dimension for the comparison judgment (e.g. “Think about the width of your own left hand”). For each trial (Fig. 1b), the fixation dot (1000 ± 250 milliseconds) was followed by a line, presented at the center of the screen. Participants were instructed to respond with the left arrow key for “shorter” and with the down key for “longer” using their right index and middle fingers respectively. The button press defined the end of the trial and was followed by a new fixation dot. The order of body parts was counterbalanced among participants. To minimize interference among the different body parts serving as reference, blocks were arranged such that those referring to judgments of the length and width of a given reference were presented consecutively, but their order was counterbalanced across blocks and participants. Participants’ hands were concealed by a white opaque panel (40 × 40 cm) while they performed the task. Participants performed the task on a quiet and dimly lit room and were seated at 55 cm distance from the monitor.

In the first study, we investigated the metric representation of the hand and foot. Before the beginning of the study we measured the width of the subject’s left hand and the length of the middle finger, as well as the width of the subjects’ left foot and the length of the big toe. Measurement processed with the subjects’ eyes closed. The length and width of the hand were 8.383 ± 0.764 and 7.700 ± 0.622 cm, respectively. The length and width of the foot were 4.577 ± 0.590 and 8.733 ± 0.723 cm. The experiment consisted of 4 blocks, each containing 80 trials. Line length ranged from −30% to +10% of the actual length of the participant’s body part with an interval of 1%. For each interval, two lines were presented. These ranges were chosen from a pilot study where we observed that both the width and the length of the hand were systematically underestimated i.e., participants responses were asymmetrically distributed, with a higher frequency of judgments for underestimation than for overestimation (Supplementary Information 3). We evaluated whether introducing an asymmetry in the range of the lines presented as stimuli could alter the underlaying estimation (e.g. by yielding a greater underestimation), by comparing the magnitude of the misestimation of the hand dimensions measured in a pilot study (N = 17) using a symmetrical range (−30 to +30%) with the asymmetrical range used in the present study (Supplementary Information 3). As no differences were observed across studies, we used the asymmetrical range in order to increase sensitivity by increasing the number of trials per condition.

The second experiment considered three body parts: the nose, the lips and the dorsal portion of the neck. The length and width of each body part were measured while the participants’ eyes were closed. Dimensions of the lips were measured while asking subjects to relax them. The width of the lips was measured from the right to the left commissures; their length was measured from the upper to the lower vermilion’s borders at the body midline. The width of the nose was measured at the naris level; its length was measured from the tip to the nasion. The width of the dorsal portion of the neck was measured from the right to the left boundary to the height of its midpoint; its length was measured as the distance between the hairline and the shoulder line. The length and width dimensions were 1.362 ± 0.230 and 4.793 ± 0.274 cm for the lips, 5.062 ± 0.396 and 3.237 ± 0.444 cm for the nose, and 8.256 ± 1.106 and 8.631 ± 0.626 cm for the dorsal portion of the neck, respectively. The second experiment consisted of 6 blocks, each containing 120 lines, ranging from −30% to +30% of the actual length of the participant’s body part with an interval of 1%.

Participants completed 10 practice trials before the beginning of the main task. The experiment was controlled and the stimuli were presented using PsychoPy^40,41^. The instructions and stimuli were presented in black ink on a grey background. Figure 1 depicts the 5 different body parts used as references and sample sizes for each experiment (a) and the line length judgement task (b).

### Analysis

Experiment 2 confirmed the general tendency to systematically underestimate the target’s dimensions (Supplementary Information 3). We thus selected for both studies trials with line distortions in the range of −30 to 10%. Responses in the LLJ task were grouped in four ranges: (i) −30 to −21%, (ii) −20 to −11%, (iii) −10 to −1%, (iv) 1 to 10%, and labels −2, −1, 0, 1 were assigned to each range, respectively. For each range, we computed the sum of “longer” responses, and used those values to fit a psychometric function to estimate the Point of Subjective Equality (PSE) separately per participant, body part and dimension. Psychometric functions were fitted using the Palamedes Toolbox (v1.9.0)^42^ in MATLAB^43^. We used the cumulative normal function to obtain the threshold and slope estimates per subject. The PSE corresponds to the line range perceived as having the same size as the reference. When the PSE = 0, the reference is veridically perceived i.e., same size as its actual dimension. A PSE > 0 indicates that the reference is perceived as larger than its actual size i.e., it is overestimated. A PSE < 0 indicates that the reference is perceived as smaller than its actual size i.e., it is underestimated. Subsequently, for each subject, body part and dimension, the PSE (ranging from −2 to 1) was transformed back into its original scale (−30 to +10%), which represents the estimation error (EE), meant as the percentage of underestimation (negative sign) or overestimation (positive sign) of a certain body part and dimension. As an example, if the PSE = −1.129, the corresponding PSE would be −11.29%, which, if referred to the estimation of a body part 8.20 cm wide, would result in a perceived size of 7.27 cm, thus in an absolute underestimation of 0.93 cm. The EE distributions were inspected for normality (Kolmogorov-Smirnov normality test) and outliers (box-and-whiskers plots). Extreme outliers were removed (higher than the third quartile plus three times the third interquartile, or lower than the first quartile minus three times the first interquartile). Seven out of 37 observations were excluded from the analyses as extreme outliers. None of the distributions violated the normality assumptions (all ps > 0.05), except for the distribution of the EEs relative to the hand width (D(14) = 0.293, p = 0.002).

To study the distortions both for the length and width of each body part separately we conducted one-sample t-tests on the EE values against zero. To study the differential effect for each dimension we also conducted repeated-measures ANOVAs on the EEs with factors body part and dimension (length/width) in SPSS^44^. In addition, we report Bayes factors - BF_10_ -^45^ which represent the likelihood of the presence of the effect (H1_1_) to the likelihood of the absence of such effect (H0), given the data. BF_10_ values larger than 1 represent evidence for the alternative hypothesis (H1). Bayesian analyses were performed in JASP^46^.

To directly compare the perceived dimensions of the body parts tested across the two studies i.e., hand, foot, lips, nose and the neck, we pooled all the EE data and analysed them by means of Linear Mixed-Effects Model in SPSS, with body parts and dimensions as fixed effects, including random intercepts and slopes to account for inter-subject variability.

As a further step, we aimed at comparing body parts as a whole, collapsing over the width and length dimensions. To this end, we used a multidimensional scaling approach: given a matrix of interpoint distances, multidimensional scaling allows to summarize the distances (or dissimilarities) among different instances and to represent them in an N-dimensional space. Concretely, we applied multidimensional scaling in MATLAB on the Euclidean distances computed among the EEs referred to the length, the width, the shape (width/length*100) and the size (length*width) of the hand, foot, lips, nose and the neck, to obtain a 2D configuration matrix.

Finally, on the pooled dataset we investigated the effect of the tactile acuity and the actual size of each body part on the EE for each dimension separately. To this end, we extracted the two-point discrimination thresholds from studies investigating tactile acuity by means of a two-point discrimination task^18–22,25^. We also explored the modulation of the effect of tactile acuity by that of the actual size, following previous reports^2,4^. For each dependent variable (length EE and width EE) we fitted four different models: (1) a null Linear Mixed Effects Model including random intercepts; (2) a model including the actual dimension as fixed effect and random intercepts and slopes; (3) a model with the tactile acuity as fixed effect including random intercepts and slopes, (4) a model with the tactile acuity, the actual dimensions as well as their interaction as fixed effects including random intercepts and slopes. We used the likelihood ratio test to detect a significant difference in the residual sum of squares between each pair of nested models. This last set of analyses were computed in R^47^.

## Supplementary information

Supplementary Information
