# Supplementary material for: Visual and somatosensory information contribute to distortions of the body model
Source: Sci Rep. 2019 Sep 19;9:13570. doi: 10.1038/s41598-019-49979-0 (PMC6753068; doi:10.1038/s41598-019-49979-0)
Supplement: Supplementary file 1 — Supplementary Information [file 41598_2019_49979_MOESM1_ESM.pdf]

SUPPLEMENTARY INFORMATION

Title:

Visual and somatosensory information contribute to distortions of the body model

Authors:

Peviani, Valeria*[1,2]; Melloni, Lucia[§]*[2,3]; Bottini, Gabriella[§1,4,5]

Affiliations:

[1] Department of Brain and Behavioural Sciences, University of Pavia, Via Bassi, 21, 27100, Pavia, Italy

[2] Department of Neuroscience, Max Planck Institute for Empirical Aesthetics, Grüneburgweg 14, 60322, Frankfurt am Main, Germany

[3] Department of Neurology, New York University School of Medicine, 240 East 38th St 10016, New York, NY, USA.

[4] Cognitive Neuropsychology Center, ASST Grande Ospedale Metropolitano Niguarda, Piazza dell'Ospedale Maggiore 3, 20162, Milan, Italy

[5] NeuroMi, Milan Center for Neuroscience, Milan, Italy

* Corresponding Authors: valeria-carmen.peviani@ae.mpg.de, lucia.melloni@ae.mpg.de

[§] These authors contributed equally to this work

Supplementary information 1

The dissimilarity matrix obtained from the multidimensional scaling analysis considering the two orthogonal dimensions for each body part.

	**Hand**	**Foot**	**Lips**	**Nose**	**Neck**
**Hand**	0	0.092	0.283	0.995	1.056
**Foot**	0.092	0	0.315	1.062	1.121
**Lips**	0.283	0.315	0	0.772	1.239
**Nose**	0.995	1.062	0.772	0	1.481
**Neck**	1.056	1.121	1.239	1.481	0

Supplementary information 2

The results for each Linear Mixed-Effects Model aimed at exploring the effects of the tactile acuity, the actual dimension and their interaction on the EEs are reported for each dimensions (Dim.). Regarding the width, the first set of models considered all the data, whereas in the second set of models the data referring to the nose representation are excluded.

Dim.	Data-set	Model	Fixed effects	Random intercepts and slopes	Log-likelihood	Likelihood ratio test statistics (against null model)
Length	All the data	0 (null)		Subjects	-111.140	/
		1	Actual length	Subjects, Actual length	-109.942	2.397, p=.494
		2	Tactile acuity	Subjects, Tactile acuity	-104.350	13.580, p=.003
		3	Tactile acuity*Actual length	Subjects, Tactile acuity*Actual length	-102.755	16.769, p=.158
Width	All the data	0 (null)		Subjects	-107.303	/
		1	Actual width	Subjects, Actual width	-100.424	13.757, p=.003
		2	Tactile acuity	Subjects, Tactile acuity	-104.856	4.914, p=.178
		3	Tactile acuity*Actual length	Subjects, Tactile acuity*Actual length	-97.848	18.909, p=.091
Width	Nose excluded	0 (null)		Subjects	-73.615	/
		1	Actual width	Subjects, Actual width	-73.496	.238, p=.971
		2	Tactile acuity	Subjects, Tactile acuity	-73.204	.821, p=.844
		3	Tactile acuity*Actual length	Subjects, Tactile acuity*Actual length	-71.877	3.476, p=.991

Supplementary information 3

Panel a) Asymmetry in the response accuracy has been observed in a pilot study investigating the hand perceived length and width in a sample of 20 participants (17 females). Each participant underwent two blocks (one for the length and one for the width) of 120 trials each, with lines that covered a distortion range from -30% to -1% (shorter) and from 1% to 30% (longer) of the actual hand dimension. To be noted that the inaccurate responses referred to shorter lines were always reflecting an underestimation of the hand dimension, whereas the inaccurate response referred to longer lines were always representing an overestimation of the hand dimension.

In order to ascertain that the misestimation of a body part distance is not significantly influenced by the range of line presented as stimuli (symmetric centered at 0 % or asymmetric), we compared the magnitude of the misestimation (Estimation Error, see Analysis section) of the hand dimensions measured in the pilot experiment with that measured in the present study. The paired t-tests on the hand length EE (pilot experiment mean EE = -.766 ± .651 vs current experiment mean EE = -.996 ± .651; t (29) = -.827, p = .415, BF_{10} = 0.443) and on the hand width EE (pilot experiment mean EE = -.380 vs current experiment mean EE = -.593; t (29) = -.785, p=.439, BF_{10} = 0.432) were both not significant.

Panel b) Response accuracy for each line distortion range is depicted for the second experiment assessing the mental representation of the nose, the lips and the rostral portion of the neck. The asymmetry observed in the pilot data regarding the hand is evident in the data referred to other body parts.

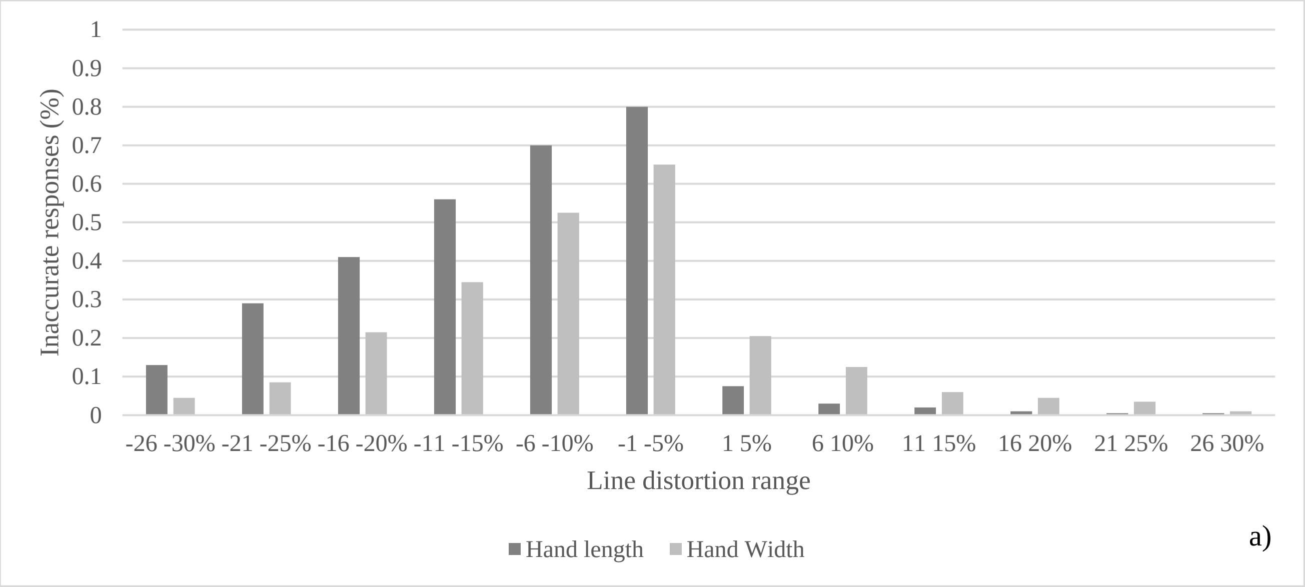

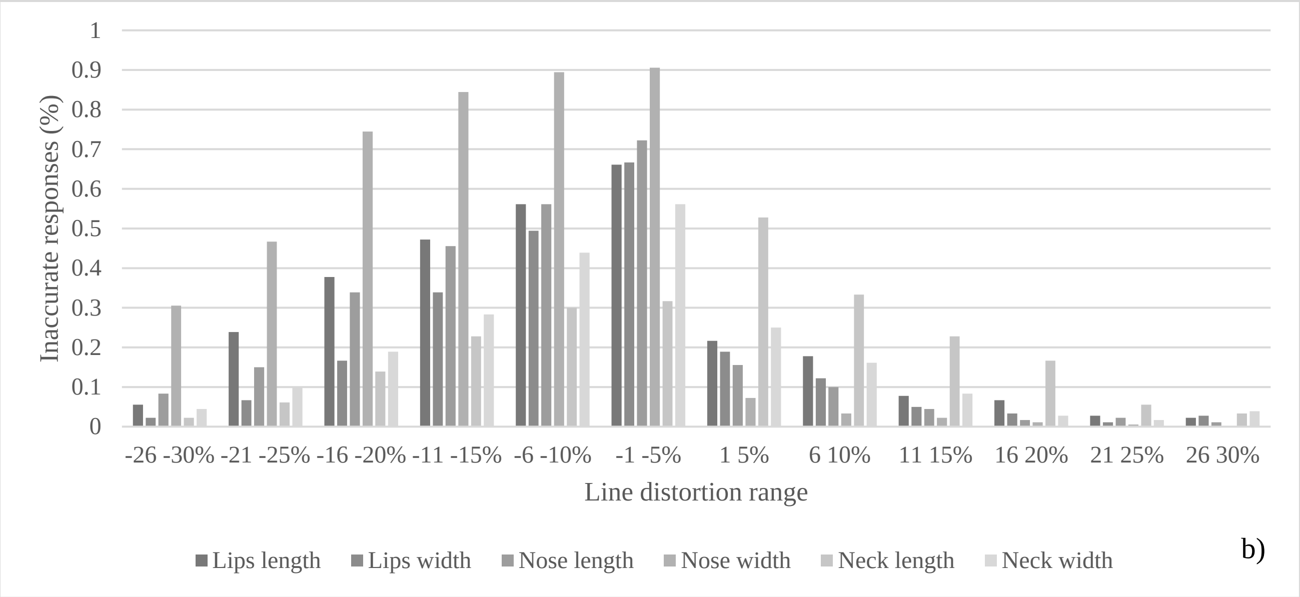

1
0.9
0.8
0.7
0.6
0.5
0.4
0.3
0.2
0.1
0
Inaccurate responses (%)
-26 -30%
-21 -25%
-16 -20%
-11 -15%
-6 -10%
-1 -5%
1 5%
6 10%
11 15%
16 20%
21 25%
26 30%
Line distortion range
Lips length
Lips width
Nose length
Nose width
Neck length
Neck width
b)
